# Geo-temporal vehicular environmental sensing dataset

**DOI:** 10.1016/j.dib.2026.112672

**Published:** 2026-03-10

**Authors:** Carmine Colarusso, Marco Consales, Gregorio Dalia, Ida Falco, Lorenzo Goglia, Francesco Cosimo Mazzitelli, Pio Antonio Perugini, Eugenio Zimeo

**Affiliations:** University of Sannio, Department of Engineering, via Traiano 3, 82100, Benevento, Italy

**Keywords:** Urban air monitoring, Pollution, Spatio-temporal data, Air quality

## Abstract

This dataset contains georeferenced, timestamped environmental measurements of CO₂ concentration and air temperature collected by a fleet of mobile sensing platforms operating in the urban area of Benevento, Italy. The data were acquired using low-power, vehicle-mounted sensor nodes designed to capture fine-grained spatio-temporal variability across the city. Each sensing unit integrates a Sensirion SCD41 nondispersive infrared CO₂ sensor, connected to an embedded computing board responsible for local preprocessing, GPS acquisition, timestamp synchronization, and data transmission.

Measurements were performed using three vehicles equipped with the same sensing hardware. The sensing fleet traversed the urban environment along diverse, randomly selected roads. All vehicles generated continuous timestamped and georeferenced (latitude, longitude, altitude) streams of CO₂, temperature, humidity, and speed measurements.

The dataset includes raw sensor readings, spatial coordinates, temporal metadata, and vehicle identifiers, enabling multi-vehicle trajectory reconstruction and environment-driven analyses.

This dataset is intended to support research on urban mobile sensing, spatio-temporal analysis, environmental monitoring, and vehicular sensor networks. Potential reuse includes benchmarking interpolation algorithms, validating mobile sensing strategies, and studying mobility-aware environmental sampling. The dataset may also be used to investigate sensor behavior in real-world dynamic conditions or to develop methods for robust, resource-aware urban monitoring.

Specifications Table

**Table:** 

Subject	Computer Sciences
Specific subject area	Distributed urban sensing for Smart City environmental monitoring using mobile vehicular data
Type of data	Table (.csv format)
Data collection	Data were collected using mobile environmental sensing units mounted on vehicles. Each unit included a Sensirion SCD41 (NDIR-based) for CO₂, humidity, and temperature, a GNSS receiver for geolocation, and a compute controller (Raspberry Pi) handling data acquisition, preprocessing, and communication. Vehicles moved along randomly selected urban routes; faulty or non-georeferenced records were removed.
Data source location	The data were collected in the urban area of Benevento, Italy, with the support of the University of Sannio. All measurements fall within a bounding box defined by the upper-left coordinates [41.143083° N, 14.760023° E] and the lower-right coordinates [41.114967° N, 14.803562° E]. The monitored area covers approximately 11.37 km².
Data accessibility	Repository name: Zenodo Data identification number: 10.5281/zenodo.17909869 Direct URL to data: https://zenodo.org/uploads/17909869
Related research article	C. Colarusso, M. Consales, I.Falco, E.Zimeo, Mobile Urban Sensing: Spatio-Temporal Observability Analysis and Optimization. https://doi.org/10.1016/j.iot.2026.101907

## 1 Value of the Data

• These data provide high-resolution spatio-temporal measurements of CO₂ concentration and temperature collected from mobile sensing platforms in an urban environment. They are valuable for understanding the variability of air quality across both space and time, supporting urban environmental research and smart city applications.
• The dataset enables benchmarking and validation of spatio-temporal interpolation and reconstruction methods. Researchers can test algorithms for mobile sensor data fusion, gap filling, and map generation under realistic urban mobility and sampling constraints.
• The dataset follows an Opportunistic Mobile Sensing (OMS) paradigm, where spatial coverage is determined by the everyday trajectories of urban service fleets (e.g., door-to-door waste collection vehicles) rather than coverage-optimized paths. Therefore, acquired data exhibit high spatial and temporal variability across narrow residential streets and main roads. This makes this dataset useful for testing the robustness of spatial-temporal interpolation methods.
• By providing georeferenced measurements across multiple opportunistic vehicle trajectories, the data support studies on mobility-aware sensing strategies, including optimizing sensing fleet dimensioning, and sensor deployment strategies.

## 2 Background

Traditional fixed sensor networks for air quality monitoring can be costly, spatially sparse, and temporally limited [1]. Opportunistic Mobile Sensing (OMS) using a fleet of service or utility vehicles (e.g., door-to-door waste collection trucks) offers a flexible and cost-effective alternative for collecting environmental data across urban areas [2]. OMS is a passive data-collection technique that uses sensors embedded in smartphones, vehicles, or wearable devices without active user involvement. It leverages user mobility to gather large-scale environmental data without predetermined mobility patterns or coverage analysis.

To systematically study the capabilities and limitations of mobile fleets, we equipped vehicles with CO₂ and temperature sensors and designed measurement campaigns covering diverse random urban routes. The dataset captures georeferenced, timestamped measurements, enabling spatio-temporal analyses and the development of a method for interpolation and coverage assessment exploiting fleet-dependent opportunistically acquired data.

This data article supports previous research on Edge-Fog-Cloud spatio-temporal interpolation frameworks [3] by providing a publicly documented dataset for multiple vehicles over a larger area. The dataset allows researchers to explore real-world challenges such as irregular sampling, fleet-dependent coverage, and communication constraints in urban settings. It provides the foundation for reproducible experiments in mobile environmental sensing, distributed data processing, and smart city monitoring.

## 3 Data Description

The dataset under the form of a Table saved in .csv format, contains environmental monitoring data collected by three vehicles equipped with the sensing device. Data acquisition took place in the city of Benevento on November 26, 2025, within the time window from 2025–11–26 10:37:41.160000+00:00 to 2025–11–26 12:27:39.022000+00:00, corresponding to a total duration of approximately 1 hour, 49 min, and 58 s. During this period, a total of 12,610 samples were recorded. All these samples are grouped in a table and saved into a file in Comma Separated Value (.csv) format.

Each table row represents an environmental observation related to a specific time and position, as in Table 1. Each observation related data are presented in columns that could be listed as follows:

• Timestamp: date and time of the recorded measurement, stored in ISO 8601 format with time zone information in UTC.
• Node: identifier of the sensing unit or vehicle that generated the sample.
• Latitude: geographic latitude coordinate of the vehicle at the time of measurement, expressed in decimal degrees.
• Longitude: geographic longitude coordinate of the vehicle at the time of measurement, expressed in decimal degrees.
• Speed: instantaneous speed of the vehicle at the moment of acquisition, measured in meters per second (m/s).
• Altitude: elevation above sea level at the sampling location, expressed in meters.
• CO2: concentration of carbon dioxide measured by the sensor, expressed in parts per million (ppm).
• Temperature: ambient air temperature measured by the sensing device, expressed in degrees Celsius (°C).
• Humidity: relative humidity of the surrounding environment, expressed as a percentage (%).
• CO2_ewma: exponential weighted moving average of CO2 concentration.
• Temperature_ewma: exponential weighted moving average of temperature readings.
• Humidity_ewma: exponential weighted moving average of relative humidity values.

**Table 1:** Example subset from the dataset.

Timestamp	Node	Latitude	Longitude	Speed	Altitude	CO2	Temperature	Humidity	CO2_ewma	Temperature_ewma	Humidity_ewma
2025–11–26T12:03:02.325000+00:00	1	41.13017	14.78252	6.026368	198.9	530	11.06804	77.42004	512.9099	12.17067	75.12628
2025–11–26T12:03:03.064000+00:00	3	41.12128	14.76921	0.96	183.7	374	11.68488	71.9635	432.9469	11.60745	73.97989
2025–11–26T12:03:03.422000+00:00	1	41.13011	14.78253	6.167916	198.9	530	11.06804	77.42004	513.2056	12.15159	75.16597
2025–11–26T12:03:04.063000+00:00	3	41.12127	14.76922	0.52	183.7	384	11.84509	71.64764	432.3496	11.61035	73.95143
2025–11–26T12:03:04.525000+00:00	1	41.13006	14.78253	5.804008	198.9	530	11.06804	77.42004	513.4949	12.13293	75.2048
2025–11–26T12:03:05.069000+00:00	3	41.12126	14.76922	1.19	183.7	384	11.84509	71.64764	431.76	11.61321	73.92334
2025–11–26T12:03:05.425000+00:00	1	41.13001	14.78253	6.099458	198.9	530	11.06804	77.42004	513.7772	12.11471	75.24269
2025–11–26T12:03:06.064000+00:00	3	41.12125	14.76923	2.13	183.7	384	11.84509	71.64764	431.178	11.61604	73.89561
2025–11–26T12:03:06.320000+00:00	1	41.12996	14.78253	5.992911	198.9	530	11.06804	77.42004	514.0529	12.09693	75.27968
2025–11–26T12:03:07.033000+00:00	2	41.13527	14.78696	10.27	169.3525	487	10.81436	78.43475	501.8866	11.4926	77.32376
2025–11–26T12:03:07.092000+00:00	3	41.12122	14.76925	2.78	183.7	384	11.84509	71.64764	430.6033	11.61883	73.86822
2025–11–26T12:03:07.381000+00:00	1	41.12992	14.78254	4.923318	198.9	533	11.01997	77.91748	514.3732	12.07872	75.32429

## 4 Experimental Design, Materials and Methods

The data were collected using a fleet of three urban vehicles equipped with mobile environmental sensing units. The sensing units included: *(i)* a Sensirion SCD41 sensor (NDIR-based) for CO₂, temperature and humidity measurements mounted on the rooftop of the vehicle; *(ii)* a smartphone to collect accurate geolocation via its position estimation (combining GPS and LTE data); *(iii)* a Raspberry Pi compute board for data acquisition, preprocess, and transmission.

The SCD41 (Specifications in Table 2) sensor measures CO₂ concentration by quantifying the energy absorbed by carbon dioxide molecules. Inside the measurement chamber, an infrared signal is pulsed and absorbed by CO₂ molecules. This absorption increases their vibrational energy, generating a corresponding pressure wave that is directly proportional to the CO₂ concentration: higher concentrations result in greater infrared absorption and, consequently, stronger pressure oscillations. An internal microphone detects these pressure variations, and the sensor electronics process the resulting signal to compute the CO₂ concentration. It uses automatic self-calibration (ASC).

**Table 2:** Sensirion SCD41 specifications.

	CO₂	Temperature	Humidity
Accuracy	± (40 ppm + 5 % MV)	0.8 °C	6 % RH
Response time	60 s	120 s	120 s

The GPS positions were acquired directly from smartphones, which estimate their geolocation using multi-source sensor fusion. In particular, modern mobile devices combine signals from global navigation satellite system constellations such as GPS, Galileo, or GLONASS with auxiliary information derived from cellular network cells and, when available, nearby Wi-Fi access points. This hybrid positioning approach improves accuracy, reduces time-to-fix, and enhances reliability in urban or partially obstructed environments where satellite visibility alone may be insufficient. This approach is increasingly used in modern applications involving crowdsensing [4,5] with an accuracy of a few meters (∼2 m horizontal and ∼15 m vertical), which is suitable for mobile scenarios.

Vehicles followed opportunistic urban routes within the city of Benevento, Italy. The data were collected in the urban area of Benevento, Italy, within a bounding box with the upper-left coordinates [41.143083° N, 14.760023° E] and the lower-right coordinates [41.114967° N, 14.803562° E]. The monitored area covers approximately 11.37 km². Each vehicle continuously acquired environmental data with a sampling interval of 1 s. Faulty or non-georeferenced samples were removed; for this reason, for some timeslots the dataset presents different sampling intervals.

All raw and processed data files are provided in CSV format, with each record containing timestamp, GPS coordinates, CO₂, temperature, and vehicle ID.

**Fig. 1:**
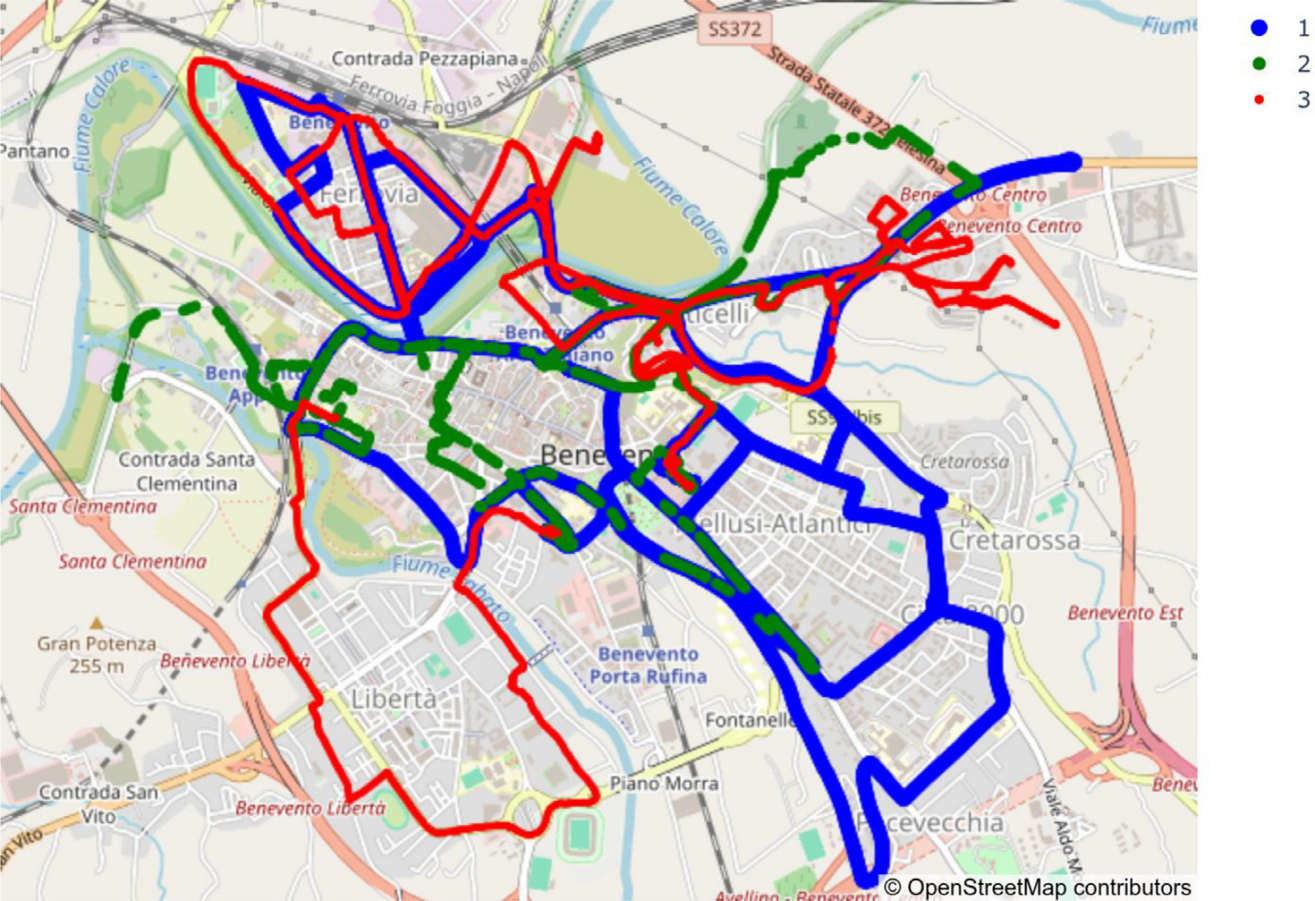
Vehicles trajectories.

Fig. 1 shows the trajectories followed by the vehicles used for environmental data collection. The route of Vehicle 1 is shown in blue, Vehicle 2 in green, and Vehicle 3 in red. In some segments, the trajectories overlap spatially, although they do not coincide in time. All vehicles were equipped with sensors utilizing active autocalibration algorithms. Although the vehicles operated in different areas, all three trajectories shared the same start and end locations. These co-located anchor points allow for a post-hoc assessment of inter-vehicle consistency. Users can treat the stationary periods at the start and end of each file as reference points to identify and correct any systematic offset effects between the vehicles.

Each vehicle contributed differently to the data collection activity. Node 1 operated for 1 hour and 22 min, covering approximately 28.73 km and collecting 4661 samples. Node 2 was active for 1 hour, 49 min, and 57 s, during which it travelled 20.29 km and acquired 3130 samples. Node 3 monitored for 1 hour, 30 min, and 11 s, covering about 23.33 km and recording 4818 samples.

Although spatial coverage has not driven this acquisition campaign, we provide post-processed coverage data in terms of traversed road classes, summarized in Table 3 and obtained via OpenStreetMap classification [6]. Points Number and Points Percentage indicate where the sensors spent most of their operational time. Almost half of the measurements (48.56 %, 6124 points) were collected along residential streets, which is consistent with typical urban monitoring dynamics. Secondary and tertiary roads together contribute roughly 33 % of the total observations, whereas primary roads (major traffic arteries) account for only 0.77 %. Coverage Percentage, instead, reflects the proportion of the total road network within each class that was traversed and sampled.

**Table 3:** Coverage analysis.

Road Class	Points Number	Points Percentage	Coverage Percentage
primary	97	0,77 %	28,60 %
secondary	1870	14,83 %	51,50 %
tertiary	2246	17,81 %	49,70 %
residential	6124	48,56 %	23,20 %
others	2273	18,03 %	9,80 %

To provide a preprocessing example aiming to reduce measurement noise and mitigate the effect of short-lived, highly localized pollution events (for example, an external vehicle in close proximity to the sensing node that briefly accelerates near the sensor, producing temporary localized emissions), an Exponentially Weighted Moving Average (EWMA) could be applied to the time series collected by each vehicle. For example, with this sensor, with a response time of 60 s for CO_2_, a time-decay EWMA with a half-life parameter of 60 s could be used. The function can be recursively applied as follows:

$$v_{k}=EWMA_{\theta_{k}}=\left\{ \begin{array}{c} x_{\theta_{k}},\;\theta_{k}=\theta_{0}\\ \alpha_{\theta_{k}}\cdot x_{\theta_{k}}+\left(1-\alpha_{\theta_{k}}\right)\cdot EWMA_{\theta_{k-1}},\;\theta_{k}>\theta_{0} \end{array}\right.$$

where the time-dependent smoothing factor $\alpha_{\theta_{k}}$ is defined as:

$$a_{\theta_{k}}=1-e^{\ln\left(\frac{1}{2}\right)\cdot \frac{\theta_{k}-\theta_{k-1}}{\Theta }}$$

In particular, in the previous equations:

• $x_{\theta_{k}}$is the value of the observation of a selected parameter at time $\theta_{k}$.
• $\theta_{0}$ is the time of the first known sample, used for the base case of the recursive function.
• $\theta_{k-1}$ is the time of the previous known observation.
• Θ controls the half-life, i.e., the time it takes for the influence of past data to halve.

**Fig. 2:**
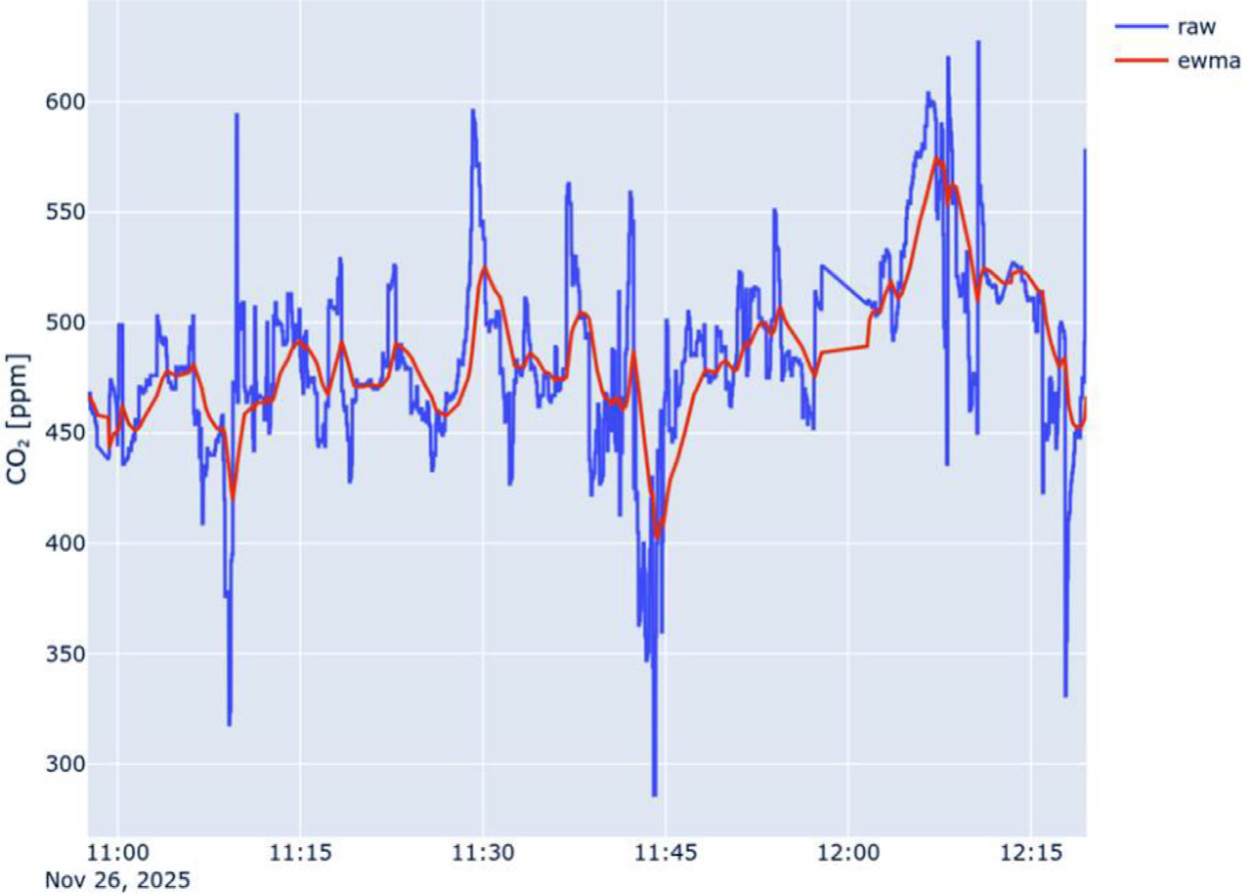
Comparison of raw and ewma-smoothed CO2 timeseries collected by Node 1.

**Fig. 3:**
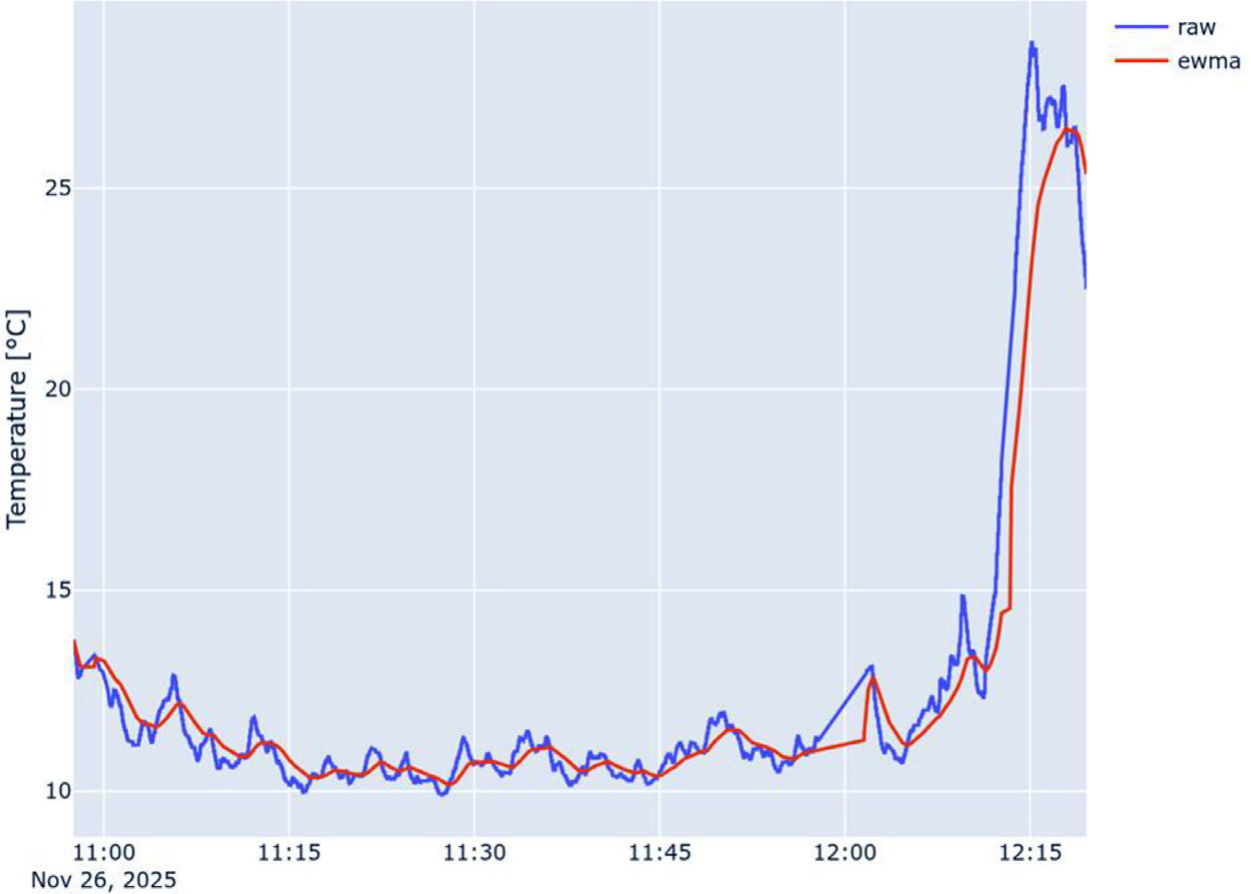
Comparison of raw and ewma-smoothed Temperature timeseries collected by Node 1.

**Fig. 4:**
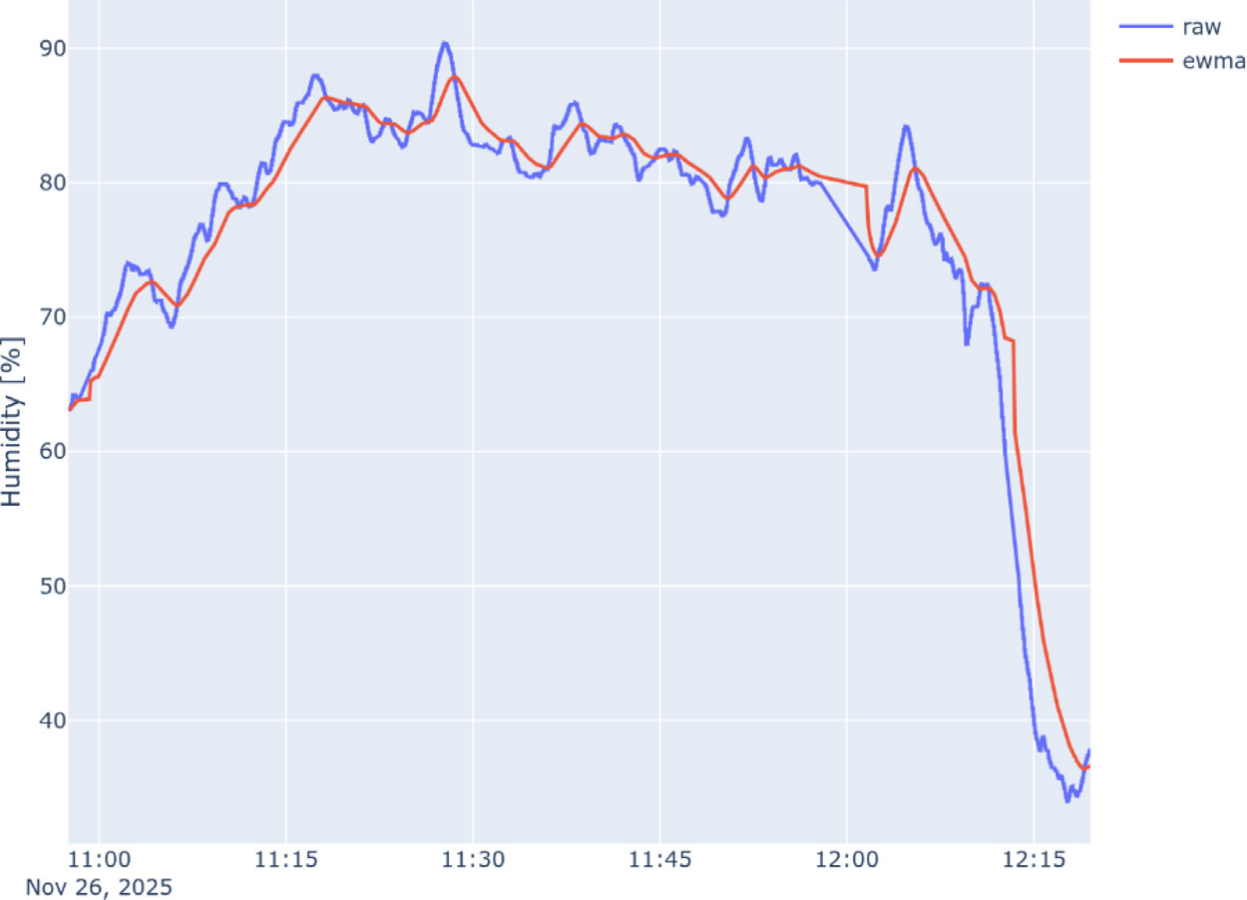
Comparison of raw and ewma-smoothed Humidity timeseries collected by Node 1.

The following Fig. 2, Fig. 3, and Fig. 4 show a comparison between the raw time series collected by the on-board sensor (in blue) and the corresponding EWMA-smoothed series (in red) with Θ=60s, the same reported in the dataset. For clarity, only the time series associated with Node 1 are reported. However, the raw measurements remain the primary reference for data reuse.

Furthermore, the abrupt signal changes visible in the final portion of the time series (Fig. 3, Fig. 4), specifically the rapid increase in temperature and the corresponding decrease in relative humidity, coincide with the final stationary co-location period to enable cross-vehicle alignment. During this phase, the vehicles were parked, and because the sensing nodes were mounted without solar radiation shields, the sensors were subject to direct solar heating.

**Fig. 5:**
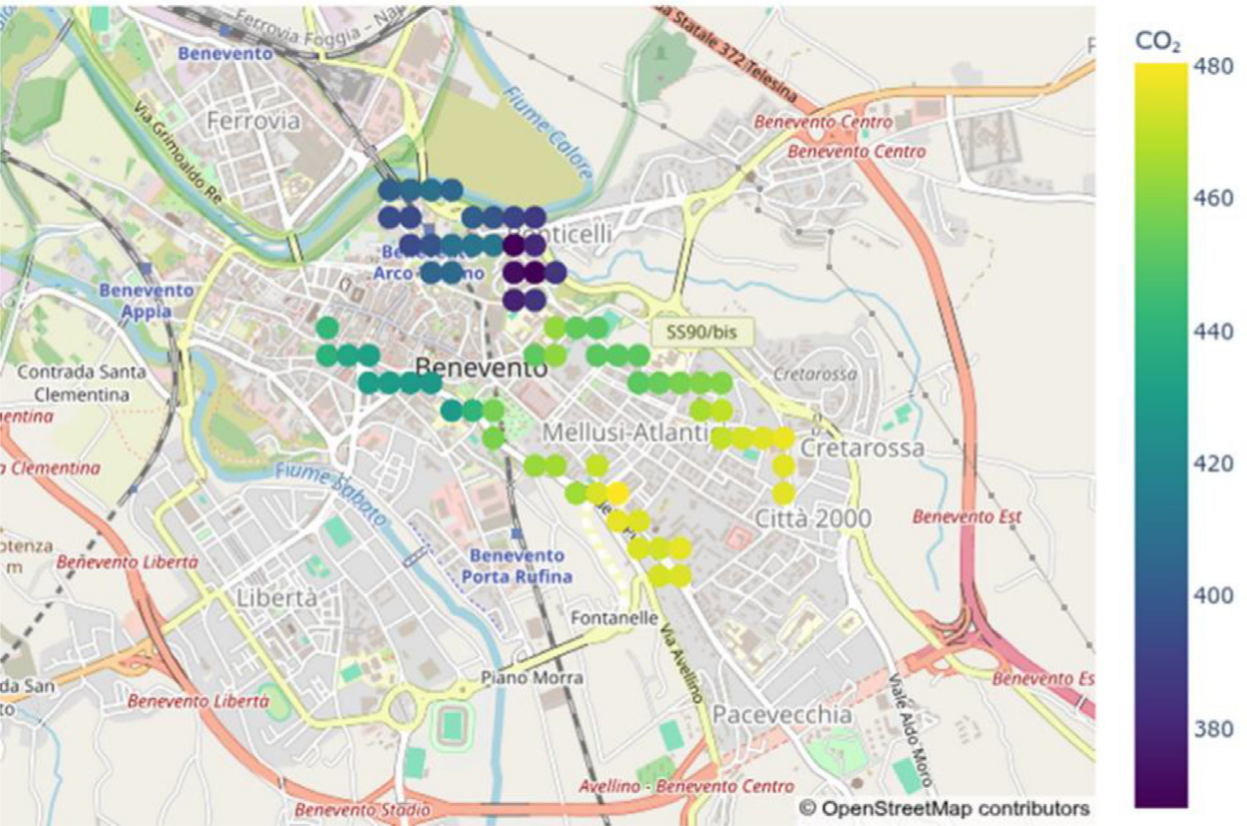
Smooted CO2 of 26/11/2025 from 11:00 to 11:05.

Finally, Fig. 5 presents a geographic heatmap of the smoothed CO₂ values over a 5-minute window (from 2025–11–26 11:00:00.000000+00:00, to 2025–11–26 11:05:00.000000+00:00), illustrating the measurements obtained simultaneously by the three nodes.

## Limitations

Environmental sensors may be subject to measurement noise or calibration drift, and occasional GPS inaccuracies can affect the precise georeference of samples.

The dataset covers a limited temporal span (approximately one hour per campaign) and a single urban area, which may restrict its representativeness for different times of day, seasons, or other cities. Although effective for opportunistic mobile sensing applications, the dataset is not intended for constructing a comprehensive environmental model.

## Ethics Statement

The authors have read and follow the ethical requirements for publication in Data in Brief and confirming that the current work does not involve human subjects, animal experiments, or any data collected from social media platforms.

## CRediT Author Statement

**Carmine Colarusso**: Conceptualization, Methodology, Formal analysis, Software, Visualization, Data curation, Writing – original draft. **Marco Consales**: Writing – review and editing, Conceptualization, Supervision, Project administration. **Gregorio Dalia**: Visualization, Data curation, Writing – original draft. **Ida Falco**: Conceptualization, Methodology, Formal analysis, Software, Visualization, Data curation, Writing – original draft. **Lorenzo Goglia**: Visualization, Data curation, Writing – original draft. **Francesco Cosimo Mazzitelli**: Visualization, Data curation, Writing – original draft. **Pio Antonio Perugini**: Visualization, Data curation, Writing – original draft. **Eugenio Zimeo**: Writing – review and editing, Conceptualization, Supervision, Project administration.

## Data Availability

SSSGeo-Temporal Vehicular Environmental Sensing Dataset (Original data).
